# Exploring the mechanism of action of licorice in the treatment of COVID-19 through bioinformatics analysis and molecular dynamics simulation

**DOI:** 10.3389/fphar.2022.1003310

**Published:** 2022-09-02

**Authors:** Jun-Feng Cao, Yunli Gong, Mei Wu, Xingyu Yang, Li Xiong, Shengyan Chen, Zixuan Xiao, Yang Li, Lixin Zhang, Wang Zan, Xiao Zhang

**Affiliations:** ^1^ Clinical Medicine, Chengdu Medical College, Chengdu, China; ^2^ Chengdu Medical College of Basic Medical Sciences, Chengdu, China; ^3^ Laboratory Medicine, Chengdu Medical College, Chengdu, China; ^4^ Yunnan Academy of Forestry Sciences, Kunming, China; ^5^ Chengdu Medical College of Pharmacy, Chengdu, China

**Keywords:** licorice, COVID-19, bioinformatics analysis, molecular docking, molecular dynamics

## Abstract

**Purpose:** The rapid worldwide spread of Corona Virus Disease 2019 (COVID-19) has become not only a global challenge, but also a lack of effective clinical treatments. Studies have shown that licorice can significantly improve clinical symptoms such as fever, dry cough and shortness of breath in COVID-19 patients with no significant adverse effects. However, there is still a lack of in-depth analysis of the specific active ingredients of licorice in the treatment of COVID-19 and its mechanism of action. Therefore, we used molecular docking and molecular dynamics to explore the mechanism of action of licorice in the treatment of COVID-19.

**Methods:** We used bioinformatics to screen active pharmaceutical ingredients and potential targets, the disease-core gene target-drug network was established and molecular docking was used for verification. Molecular dynamics simulations were carried out to verify that active ingredients were stably combined with protein targets. The supercomputer platform was used to measure and analyze stability of protein targets at the residue level, solvent accessible surface area, number of hydrogen bonds, radius of gyration and binding free energy.

**Results:** Licorice had 255 gene targets, COVID-19 had 4,628 gene targets, the intersection gene targets were 101. Kyoto Encyclopedia of Genes and Genomes (KEGG) and Gene ontology (GO) analysis showed that licorice played an important role mainly through the signaling pathways of inflammatory factors and oxidative stress. Molecular docking showed that Glycyrol, Phaseol and Glyasperin F in licorice may playe a role in treating COVID-19 by acting on STAT3, IL2RA, MMP1, and CXCL8. Molecular dynamics were used to demonstrate and analyze the binding stability of active ingredients to protein targets.

**Conclusion:** This study found that Phaseol in licorice may reduce inflammatory cell activation and inflammatory response by inhibiting the activation of CXCL8 and IL2RA; Glycyrol may regulate cell proliferation and survival by acting on STAT3. Glyasperin F may regulate cell growth by inhibiting the activation of MMP1, thus reducing tissue damage and cell death caused by excessive inflammatory response and promoting the growth of new tissues. Therefore, licorice is proposed as an effective candidate for the treatment of COVID-19 through STAT3, IL2RA, MMP1, and CXCL8.

## Graphical Abstract

The mechanisms analysis of licorice in the treatment of COVID-19.
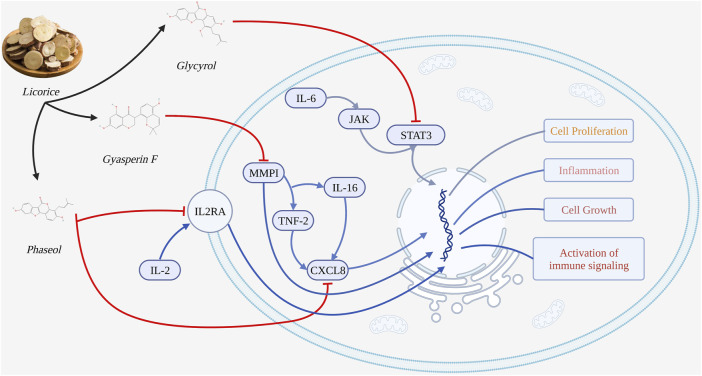

## Introduction

Corona Virus Disease 2019 (COVID-19) is a respiratory disease caused by Severe Acute Respiratory Syndrome Coronavirus 2 (SARS-CoV-2) (Fernandes et al., 2022). Signs and symptoms of COVID-19 disease vary from patient to patient, but the most common clinical signs include fever, fatigue, cough, anorexia, sputum production and shortness of breath (Rai et al., 2021). Less common symptoms such as sore throat, headache, confusion, hemoptysis, shortness of breath, and chest tightness, as well as mild symptoms such as nausea, vomiting, diarrhea, and gastrointestinal complications have also been reported (Majumder and Minko, 2021). SARS-CoV-2 transmission usually occurs via respiratory droplets with an average incubation period of 6.4 days. Although most patients tend to be mildly ill, a small number of patients develop severe hypoxia requiring hospitalization and mechanical ventilation (Ochani et al., 2021). In severe cases, pneumonia, severe acute respiratory syndrome, heart failure, renal failure, and even death occur (Rai et al., 2021). However, there is a lack of effective COVID-19 therapeutic agents with few side effects. Therefore, screening and investigating drugs to treat COVID-19 will contribute significantly to the global fight against the COVID-19 epidemic.

Clinical evidence suggests that herbal drugs are effective against viral infections such as influenza, SARS and SARS-CoV-2 by targeting viral cell entry, viral replication and host antiviral immune response steps. Among the drugs and formulations recommended by Chinese authorities for COVID-19 treatment, the dried root of licorice is one of the most commonly used ingredients in formulations. Recent reports also suggest that licorice extracts may play a potential role in the fight against COVID-19 and related diseases (Li et al., 2021). According to the Chinese Pharmacopoeia, licorice is able to nourish the spleen, remove heat, prevent toxicity, remove phlegm, and relieve cough, cramps and pain, thus harmonizing the effects of other drugs (Ng et al., 2021).

Many studies have reported that active compounds isolated from licorice have antitumor, antibacterial, antiviral, anti-inflammatory, immunomodulatory and several other activities that help restore and protect the nervous, digestive, respiratory, endocrine and cardiovascular systems (Yang et al., 2015). Licorice has many pharmacological effects and is often used as a unique “guiding drug,” accounting for more than half of the traditional and modern prescriptions and formulations. The modulating effects of licorice on other herbs include significant detoxification, treatment of drug and food poisoning, or suppression of adverse reactions, and this “guiding” effect has been tested in many preparations. According to available studies, the pharmacological effects of licorice and natural products such as glycyrrhizin have beneficial effects on the prevention of some immune reactions triggered by COVID-19 (Zhang et al., 2021). In addition to antiviral and anti-inflammatory properties, one of the components of licorice has a mechanism to enhance autophagy, which studies have shown to be necessary for COVID-19 treatment (Abraham and Florentine, 2021).

Numerous studies have been conducted to find many active components in licorice that can hinder SARS-COV-2 infection and alleviate the clinical symptoms of COVID-19. Gomaa and Abdel-Wadood demonstrated the antiviral activity of licorice sweeteners and licorice extracts. The most common mechanism of antiviral activity is due to disruption of viral uptake into host cells and disruption of the interaction between SARS-COV2 and the receptor binding structural domain (RBD) of ACE2 (Gomaa and Abdel-Wadood, 2021). Luo found that quercetin, the active component of licorice, has a strong docking ability with IL-6, suggesting that licorice may primarily reduce IL-6 levels in response to COVID-19 inflammatory outbreaks, which represents a prospective therapeutic strategy for moderate COVID-19 (Luo et al., 2022). Yi et al. found that the triterpenoid licorice saponin A3 (A3) and glycyrrhizic acid (GA) could effectively inhibit SARS-CoV-2 by targeting nsp7 and the stinging protein RBD, respectively (Yi et al., 2022). However, licorice as a traditional Chinese medicine contains a large number of active ingredients, and the complex drug composition seriously hinders the application of licorice in clinical COVID-19 treatment, and the specific mechanism of action of licorice for the treatment of COVID-19 is still unclear. Molecular dynamics allows a comprehensive and systematic simulation of the interaction and binding stability between small molecule monomers and protein targets with the help of powerful computational capabilities.

Molecular dynamics (MD) is based on large computer clusters (even supercomputers) and aims to computationally obtain data on the microstructure, physicochemical properties, and performance characterization parameters of materials (Collier et al., 2020). Molecular dynamics complements and digs deeper into the traditional materials discipline, which is mainly experimental. The data obtained from calculations are used to study and analyze the mechanism behind the experiments at multiple levels from micro, meso and macro scales (Nam, 2021). Molecular dynamics simulations help to discover the relationships on protein, protein-ligand, protein-protein, protein-DNA and other biomolecular interactions (Al-Shar’i and Al-Balas, 2019). Molecular dynamics simulations not only help to understand the physical processes of systems at the atomic level, but also allow the discovery of empirically undetectable hidden states. In addition, experimental measurements of thermodynamic properties in biomolecular systems are usually expensive and time-consuming (Filipe and Loura, 2022). Accurate theoretical calculations of their free energies by numerical simulations are becoming increasingly important in medical biology, where 3D structures of small molecule-protein complexes can reveal how and where a protein interacts with a drug small molecule.

In this study, we screened licorice for potential active small molecules by bioinformatics. The core intersection targets of licorice and COVID-19 were screened. Protein-protein interaction (PPI), Kyoto Encyclopedia of Genes and Genomes (KEGG) and Gene ontology (GO) were used to analyze the potential association among the core intersection targets to explore the mechanism of action and potential pathways. To further validate the relationship between active small molecules and key protein targets we performed molecular motion system simulations through a supercomputer platform. Molecular motion system simulations enable systematic study and analysis of drugs to treat diseases from the cellular level to the chemical moiety level. Molecular docking was used to determine the affinity of monomeric compounds to protein targets, and molecular dynamics was used to simulate the stability of bound complexes and to analyze the dynamics of complexes after binding.

Therefore, this study of the potential mechanism of licorice in the treatment of COVID-19 may provide new ideas and necessary theoretical basis for clinical treatment.

## Material and methods

### Identification and screening of active compounds of licorice

In this study, all compounds of licorice were screened and analyzed using the Traditional Chinese Medicine System Pharmacology Database (TCMSP) (Xie et al., 2021). We evaluated the drug components in terms of absorption, distribution, metabolism and excretion and screened by two key parameters, oral bioavailability (OB) and drug similarity (DL). OB largely determines the impact of drug small molecules on disease and DL is used for early screening and refinement of candidate compounds in drug development. Active compounds of licorice were screened on the basis of OB ≥ 30% and DL ≥ 0.18.

### Analysis and screening of core intersection gene targets

We used the GeneCards database and “COVID-19” and “SAR-Cov-2” were used as keywords to obtain disease gene targets. We also imported licorice into the GeneCards database to obtain drug gene targets. Drug gene targets and disease gene targets were intersected through the venny website to obtain intersecting gene targets. And the intersecting gene targets were screened to obtain core intersecting gene targets by relevance score ≥2 as a threshold, which is a comprehensive evaluation of the association of genes with the studied diseases.

### Construction of protein-protein interaction network for corona virus disease 2019 interaction in licorice treatment

The STRING database was used to analyze protein-protein interactions (PPI) for licorice treatment of COVID-19. In this study, all the core intersecting targets were imported into Cytoscape 3.7.1 for analysis in order to elucidate the interactions between potential protein targets (Pan et al., 2020). The network topology parameters were analyzed by Cytoscape 3.7.1, and the hub protein targets were screened according to the criteria of nodal degree value and median centroid value greater than the mean.

### Gene target enrichment analysis

Interacting gene targets were analyzed by Gene Ontology (GO) functional annotation and Kyoto Encyclopedia of Genes and Genomes (KEGG) enrichment in the DAVID database. In this study, the relevant biological processes (BP), cellular components (CC) and molecular functions (MF) of the gene targets were obtained by GO enrichment. The core intersecting targets were imported into the DAVID database and the selected species was “*Homo sapiens*” (Xiong et al., 2020). We performed KEGG pathway enrichment analysis for the relevant signaling pathways involved in the disease-related targets and performed gene target screening at *p* < 0.05. The main biological processes and signaling pathways were analyzed for licorice treatment of COVID-19. The Omicshare tool platform was used to visualize the results of GO enrichment and KEGG enrichment (Cao et al., 2022).

### Validation of molecular docking and docking protocols

Molecular docking was used to study the molecular affinity of the active small molecules of licorice to the COVID-19 protein target. The crystal structures of the proteins used for docking were downloaded from the PDB database and the 3D structures of the small molecules were downloaded from the PUBCHEM database. We used AutoDock Vina 1.1.2 software for the molecular docking work. Prior to docking, PyMol 2.5 was used to process all receptor proteins (Burley et al., 2017). ADFRsuite 1.0 was used to convert all processed small molecules and receptor proteins into the PDBQT format required for docking with AutoDock Vina 1.1.2. The docked conformation with the highest output score was considered to be the binding conformation for subsequent molecular dynamics simulations (Ravindranath et al., 2015). In this study, the original crystal ligand of the protein target was used as a positive reference by re-docking the original crystal ligand and the protein. The consistency of the binding pattern can indicate the correctness of the molecular docking scheme (Cao et al., 2022).

### Molecule dynamics

In this study, the small molecule-protein complexes obtained by molecular docking were used as the initial structures for all-atom molecular dynamics simulations, respectively (Mithun et al., 2022). AMBER 18 software was used for the molecular dynamics simulations (Maier et al., 2015; Lee et al., 2020). The LEaP module was used to add hydrogen atoms to the system, a truncated octahedral TIP3P solvent box was added at a distance of 10 Å from the system, and Na+/Cl-was added to the system to balance the system charge. At the maintenance temperature of 298.15 K, the NVT (isothermal isomer) system simulation was performed for 500 ps to further distribute the solvent molecules uniformly in the solvent box. The equilibrium simulation of the whole system was performed for 500 ps at NPT (isothermal isobaric). Finally, two composite systems are simulated for 50 ns of NPT system under periodic boundary conditions (Larini et al., 2007).

### MMGBSA binding free energy calculation

The binding free energy between the protein and ligand for all systems was calculated by the MM/GBSA method (Chen et al., 2020). The MD trajectory of 50 ns was used as the calculation in this study. The calculation equations ars as follows:

$$\begin{array}{l} {\Delta G}_{\text{bind}}={\Delta G}_{\text{complex}}\;–\;\left({\Delta G}_{\text{receptor}}+{\;\Delta G}_{\text{ligand}}\right)\\ ={\Delta E}_{\text{internal}}+{\Delta E}_{\text{VDW}}+{\Delta E}_{\text{elec}}+{\Delta G}_{\text{GB}}+{\Delta G}_{\text{SA}} \end{array}$$

In this formula, the non-polar solvation free energy (ΔG_GA_) was calculated based on solvent accessible surface area (SA) and the product of surface tension (γ), ΔG_GA_ = 0.0072 × SASA (Cao et al., 2022).

## Results

### Identification of potentially active compounds in licorice

The identification of potentially active compounds in licorice was based on the criteria of DL ≥ 0.18 and OB ≥ 30%. 200 potential compounds in licorice were retrieved from the TCMSP database. By further improving the OB score (OB ≥ 70%), 11 core active compounds were screened from licorice, shown in Table 1.

**TABLE 1:** The core active compounds in licorice.

MOL ID	molecule_name	OB	MW	DL
MOL002311	Glycyrol	90.77	366.39	0.66
MOL004990	7,2′,4′-trihydroxy-5-methoxy-3-arylcoumarin	83.71	300.28	0.27
MOL004904	Licopyranocoumarin	80.36	384.41	0.65
MOL004891	Shinpterocarpin	80.29	322.38	0.72
MOL005017	Phaseol	78.76	336.36	0.57
MOL004841	Licochalcone B	76.75	286.30	0.19
MOL004810	Glyasperin F	75.83	354.38	0.53
MOL001484	Inermine	75.18	284.28	0.53
MOL000500	Vestitol	74.65	272.32	0.20
MOL005007	Glyasperins M	72.67	368.41	0.59
MOL004941	(2R)-7-hydroxy-2-(4-hydroxyphenyl)chroman-4-one	71.12	256.27	0.18

### Acquisition of intersectional target genes

**FIGURE 1:**
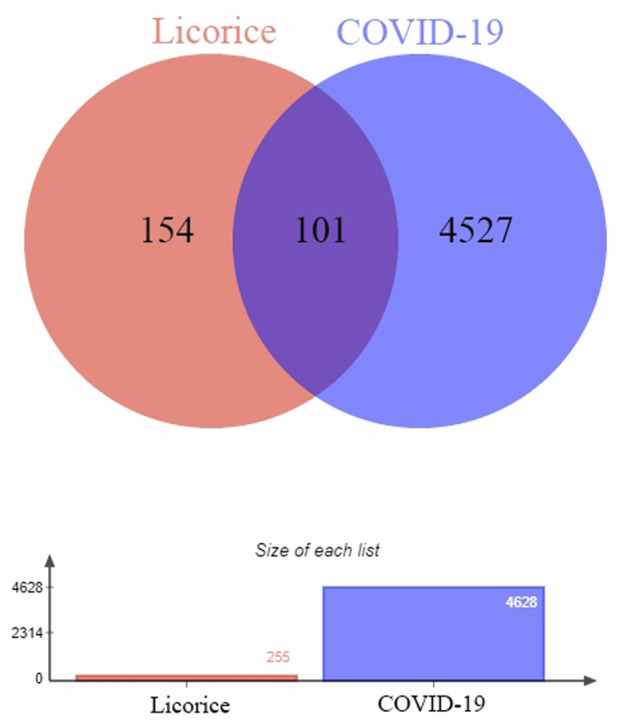
Intersection targets-active ingredient networks. Targets of the intersection of licorice and COVID-19.

In this study, 255 gene targets of licorice and 4,628 gene targets of COVID-19 were obtained. A total of 101 intersecting gene targets were processed by Venny, shown in Figure 1.

### Core intersectional target screening and protein interaction network diagram construction

**FIGURE 2:**
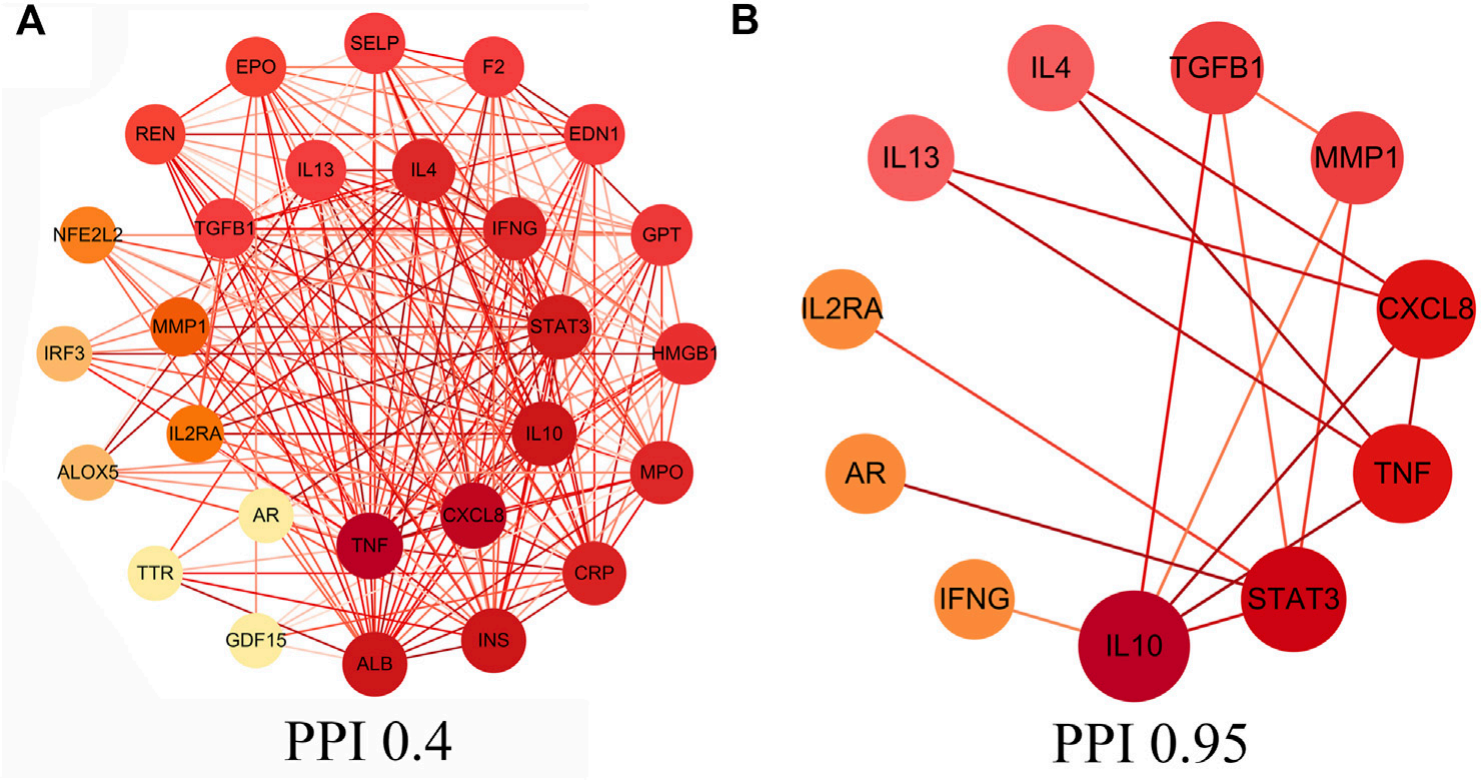
Protein-protein interaction (PPI) network. **(A)** PPI network of protein targets, **(B)** PPI network of key protein targets (confidence>0.95).

In this study, core intersectional gene targets were obtained from the GeneCards database based on relevance score, and relevance score ≥2 were considered as core intersectional gene targets. The STRING database was used to analyze the 27 core intersectional protein targets of COVID-19 and licorice, and a protein interaction network diagram was constructed for the treatment of COVID-19 with licorice, shown in Figure 2A. 11 key intersectional protein targets (such as: STAT3, IL2RA, CXCL8, etc.) were obtained by increasing the confidence score (confidence level ≥0.95), and the 11 key intersectional protein targets were used to construct the key protein interaction network diagram, shown in Figure 2B.

### Gene ontolog and kyoto encyclopedia of genes and genomes enrichment analysis

**FIGURE 3:**
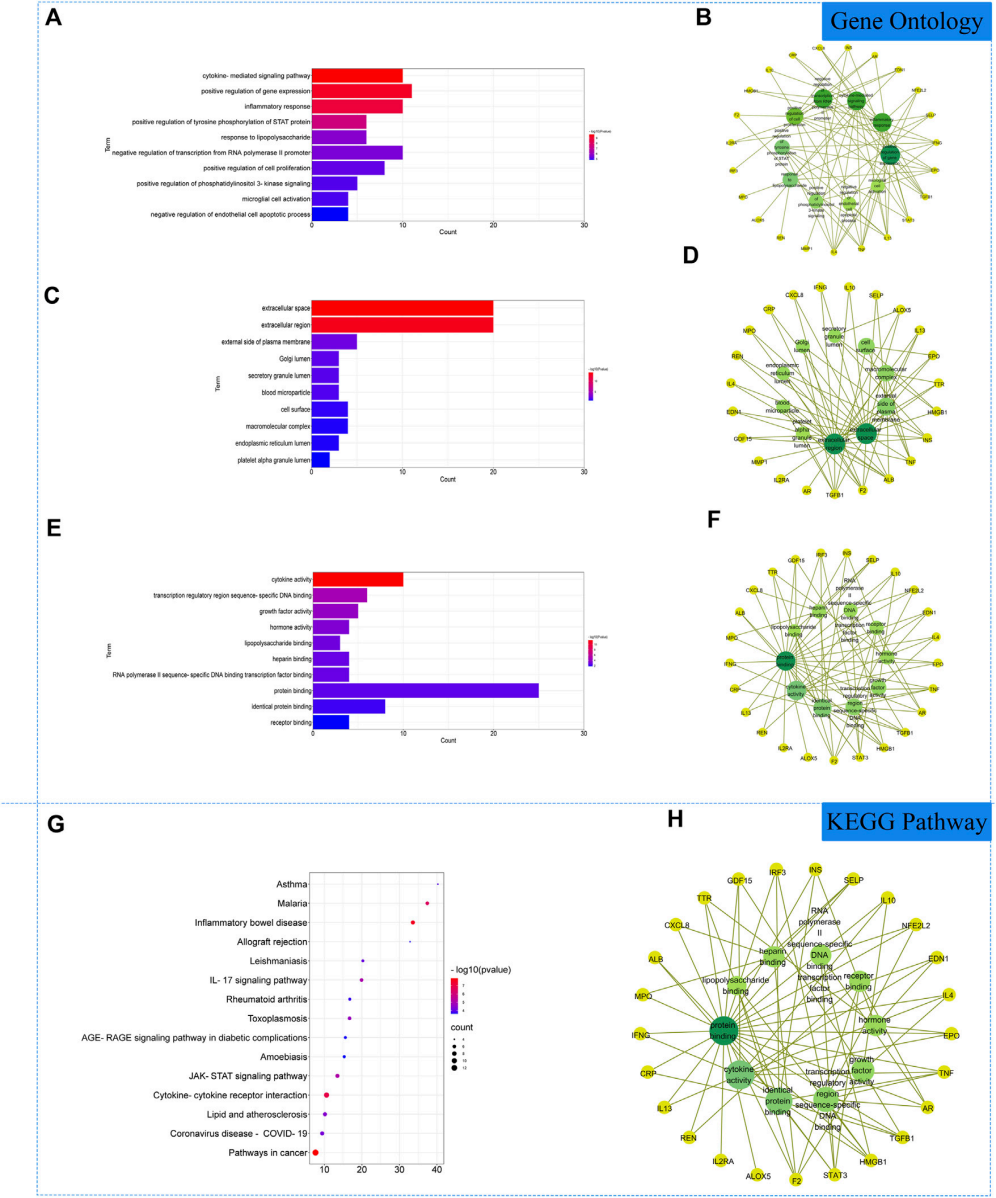
Gene Ontology (GO) and Kyoto Encyclopedia of Genes and Genomes (KEGG). Analysis of related genes. **(A)** The top 10 terms in biological processes (BP) were greatly enriched. **(B)** The subnetwork displayed the top 10 BP terms and related genes. **(C)** The top 10 terms in cellular components (CC) were greatly enriched. **(D)** The subnetwork displayed the top 10 CC terms and related genes. **(E)** The top 10 terms in molecular function (MF) were greatly enriched. **(F)** The subnetwork displayed the top 10 MF terms and related genes. **(G)** The top 15 KEGG pathways were showed. **(H)** The subnetworks displayed the top 15 KEGG pathways.

The 27 core intersectional gene targets were imported into the DAVID database for enrichment analysis. At *p* < 0.05, the GO enrichment analysis yielded 222 GO entries, including 193 BP entries, 10 CC entries and 19 MF entries. The results showed that biological processes were highly correlated with inflammation and cytokine transmission, mainly involving the positive regulation of gene expression, cytokine-mediated signaling pathway and inflammatory response. In cellular component, external side of plasma membrane, extracellular space and extracellular region account for a relatively large amount. In molecular functions, transcription regulatory region sequence-specific DNA binding, cytokine activity and growth factor activity were relatively high, shown in Figures 3A–F. KEGG pathway analysis yielded 72 pathways, and KEGG enrichment analysis showed that the enriched pathways involved multiple pathways related to immune response regulation and inflammation, mainly cytokine-cytokine receptor interaction, pathways in cancer, inflammatory bowel disease and other signaling pathways, shown in Figures 3G, H.

### Disease-core gene target-drug network

**FIGURE 4:**
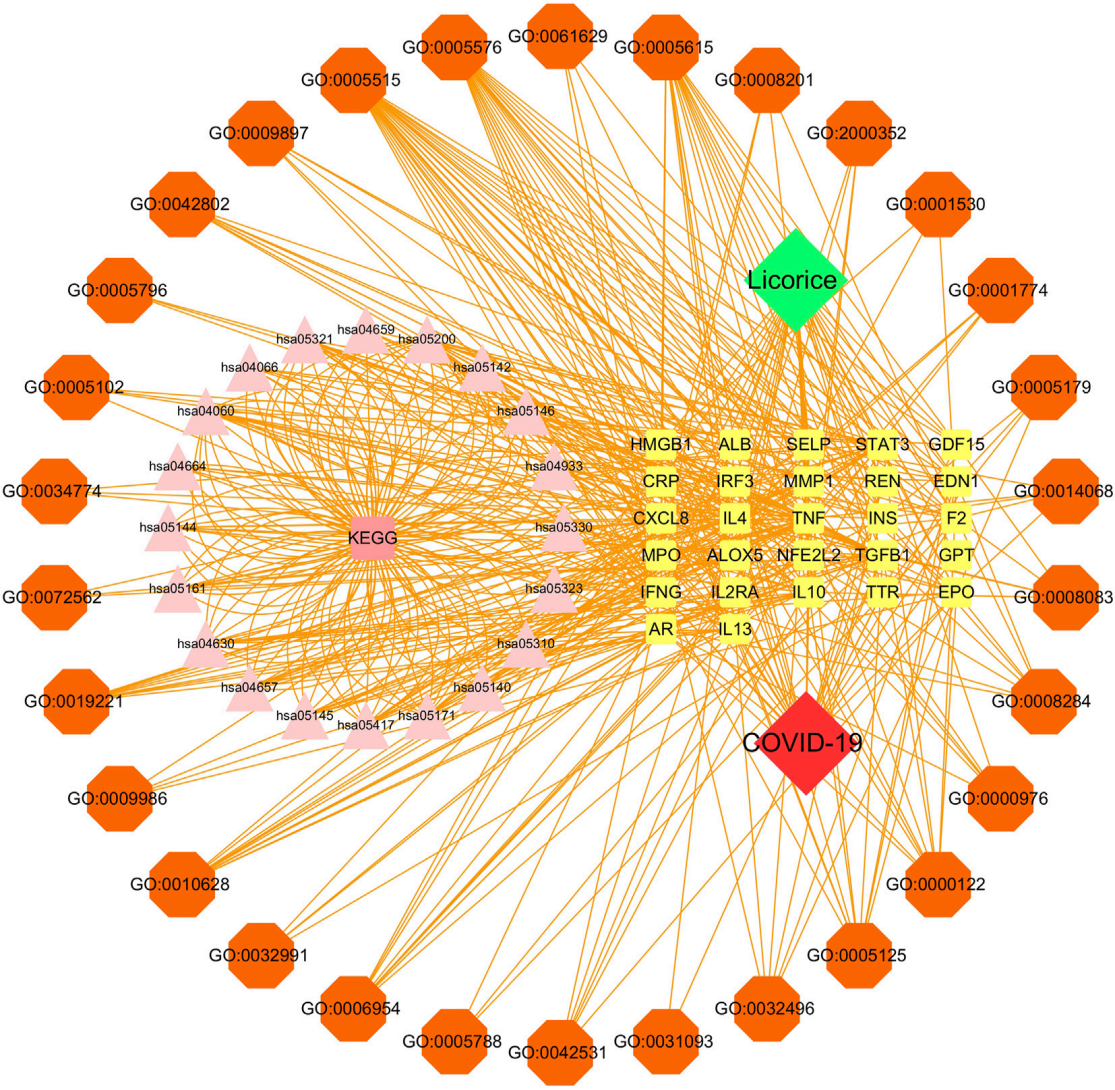
Disease-core gene target-drug network. Square nodes represent gene targets, triangular nodes represent signaling pathways (KEGG), and octagonal nodes represent gene ontology (GO) of related genes.

The disease-core gene target-drug network was constructed to demonstrate the main signaling pathways and biological processes of licorice for the treatment of COVID-19, shown in Figure 4.

### Molecular docking

**FIGURE 5:**
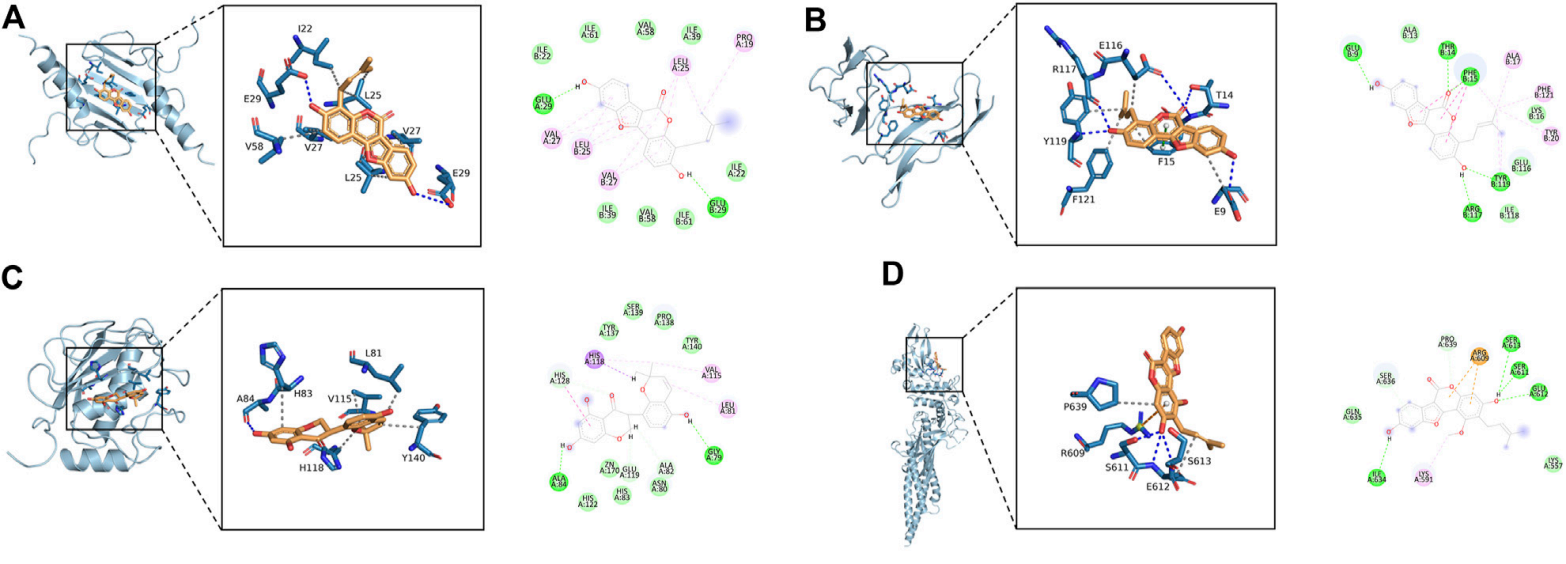
Molecular docking of active ingredients and core targets. **(A)** CXCL8/Phaseol, **(B)** IL2RA/Phaseol, **(C)** MMP1/Glyasperin F, **(D)**STAT3/Glycyrol.

**FIGURE 6:**
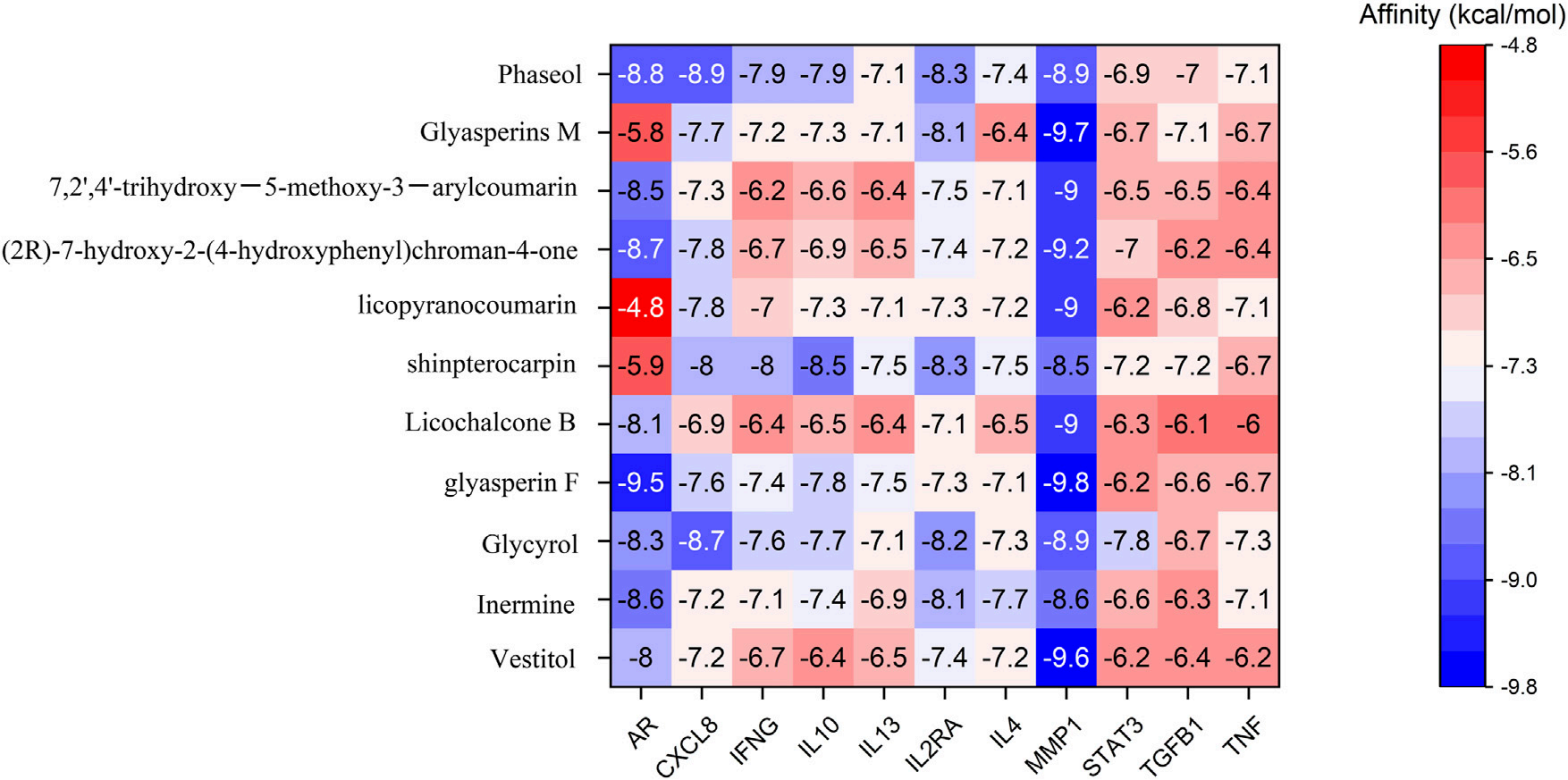
Screening docking results between ligands and receptors.

The 11 key intersection protein targets were selected for molecular docking. The results indicate that the CXCL8/Phaseol complex was mainly maintained by hydrophobic interactions. The small molecule Phaseol interacted with E29 on the protein by hydrogen bonding and with V25, V27, V58, and I22 by hydrophobic interactions, shown in Figure 5A. The binding of the IL2RA/Phaseol complex was maintained mainly by hydrogen bonding and hydrophobic interactions. The small molecule Phaseol interacted with Y119, E116, R117, T14, and E9 on the protein by hydrogen bonding and with Y119, F121, F15, E9, and E116 by hydrophobic interactions. In addition, we also observed pi-pi conjugation between Phaseol and F15, shown in Figure 5B. In the MMP1/Glyasperin F complex, the small molecule Glyasperin F interacted with A84 on the protein by hydrogen bonding and with H83, V115, L81, Y140, and H118 by hydrophobic interactions, shown in Figure 5C. The binding of STAT3/Glycyrol indicated that the small molecule Glycyrol hydrogen bonds with S611, E612, and S613 on the protein, hydrophobic interaction with P629 and S613, and also cation pi conjugation with R609, shown in Figure 5D. The molecular docking results score are shown in Figure 6.

### Molecular dynamics results

**FIGURE 7:**
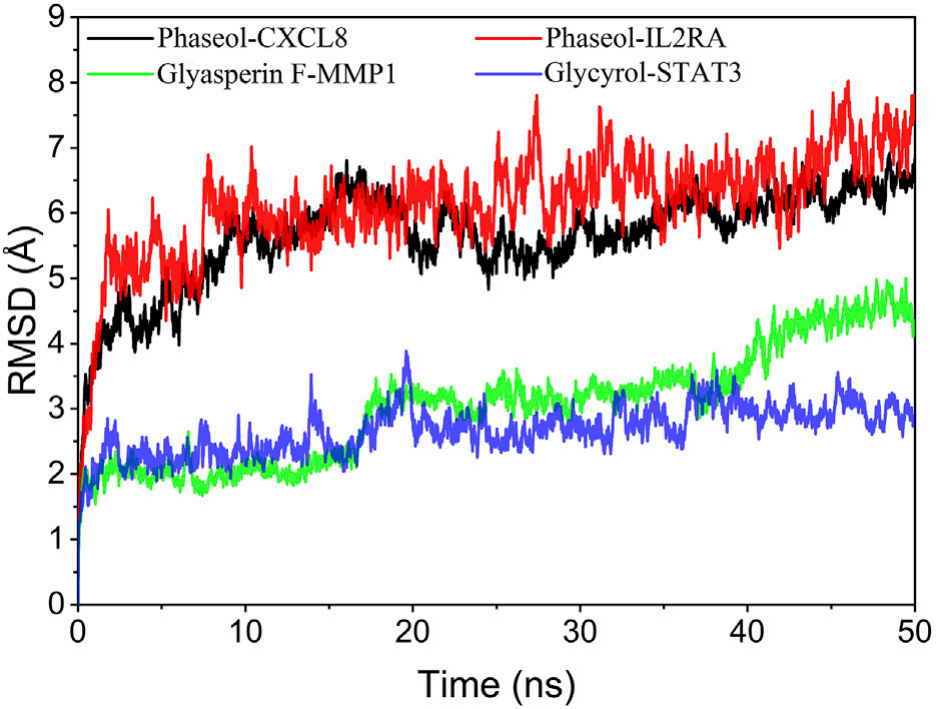
Complex root mean square deviation (RMSD) difference over time.

The root mean square deviation of the molecular dynamics simulations can reflect the motility of the complexes, and the larger RMSD and the more intense fluctuations indicate intense motility. The simulation results suggested that the RMSD fluctuations of MMP1/Glyasperin F and STAT3/Glycyrol were within 4 Å, which implied that the system was less kinetic. Therefore, combining the magnitude of RMSD and stability, we can determine the stability of these complexes from strong to weak in the order of STAT3/Glycyrol, MMP1/Glyasperin F, CXCL8/Phaseol, and IL2RA/Phaseol. The results are shown in Figure 7.

### Combined free energy calculation results

Based on the trajectory of molecular dynamics simulations, the binding energy was calculated in this study using the MM-GBSA method. The binding energy can more accurately reflect the binding mode of small molecules and target proteins. The experimental results showed that CXCL8/Phaseol, IL2RA/Phaseol, MMP1/Glyasperin F, STAT3/Glycyrol were −39.51 ± 2.06 kcal/mol, −20.12 ± 3.38 kcal/mol, −43.70 ± 1.80 kcal/mol, −11.85 ± 1.06 kcal/mol. Negative values indicate that these two molecules have binding affinity to the target protein, and lower values indicate stronger binding. The simulation results suggested that these molecules and the corresponding binding affinities are very strong. The MMP1/Glyasperin F binding energy was the highest, with a value of 43.70 ± 1.80 kcal/mol. The binding energies of these complexes were mainly contributed by van der Waals energy as well as electrostatic energy. The experimental results are shown in Table 2.

**TABLE 2:** Binding free energies and energy components predicted by MM/GBSA (kcal/mol).

System name	Δ*E*_vdw_	Δ*E*_elec_	ΔG_GB_	ΔG_SA_	ΔG_bind_
Phaseol-CXCL8	−40.63 ± 1.83	−14.59 ± 1.22	21.41 ± 1.27	−5.69 ± 0.10	−39.51 ± 2.06
Phaseol-IL2RA	−28.99 ± 2.58	−10.08 ± 9.51	22.88 ± 6.68	−3.92 ± 0.27	−20.12 ± 3.38
Glyasperin F-MMP1	−39.19 ± 1.25	−12.17 ± 2.77	11.90 ± 2.37	−4.24 ± 0.08	−43.70 ± 1.80
Glycyrol-STAT3	−14.88 ± 1.19	−1.77 ± 2.41	6.56 ± 2.24	−1.75 ± 0.14	−11.85 ± 1.06

ΔE_vdW_: van der Waals energy.

ΔE_elec_: electrostatic energy.

ΔG_GB_: electrostatic contribution to solvation.

ΔG_SA_: non-polar contribution to solvation.

ΔG_bind_: binding free energy.

### Hydrogen bond analysis

**FIGURE 8:**
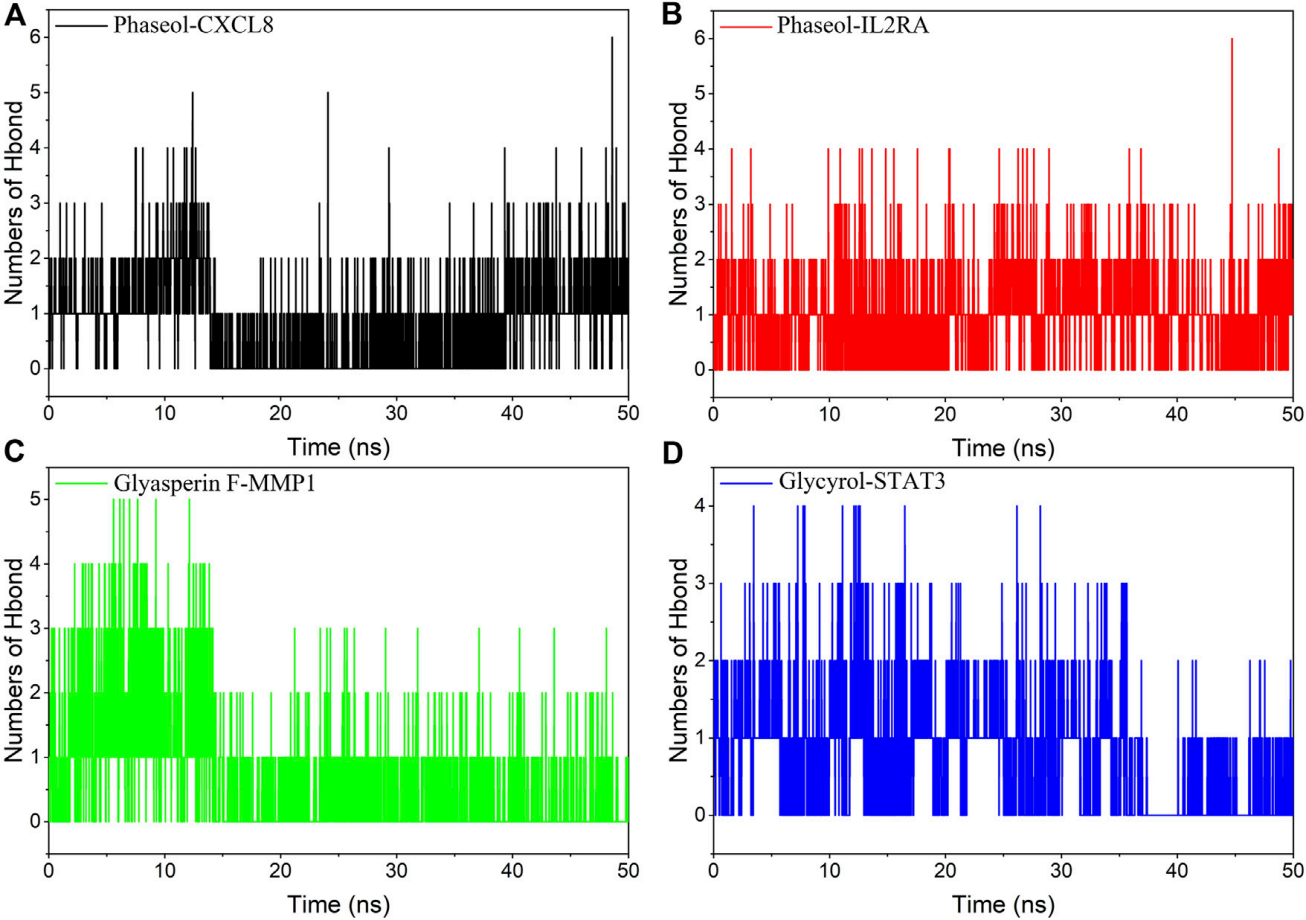
Changes in the number of hydrogen bonds between small molecule ligands and protein receptors in complex system simulations. **(A)**CXCL8/Phaseol, **(B)** IL2RA/Phaseol, **(C)** MMP1/Glyasperin F, **(D)**STAT3/Glycyrol.

Hydrogen bonding is one of the strongest non-covalent binding interactions, and a higher number indicates better binding. The experimental results suggested that the number of hydrogen bonds of the four complexes was basically 1-2 in the middle and late stages of the simulation. Among them, the hydrogen bonding diagram of MMP1/Glyasperin F complex showed more sparse in the late stage of simulation, implying that hydrogen bonding was not the main force for it to maintain stability. Combining the results of MGBSA and the binding pattern, we suggested that hydrophobic interaction was the main force for MMP1/Glyasperin F to maintain stability. The results are shown in Figure 8.

### The stability of the target protein at the residue level

**FIGURE 9:**
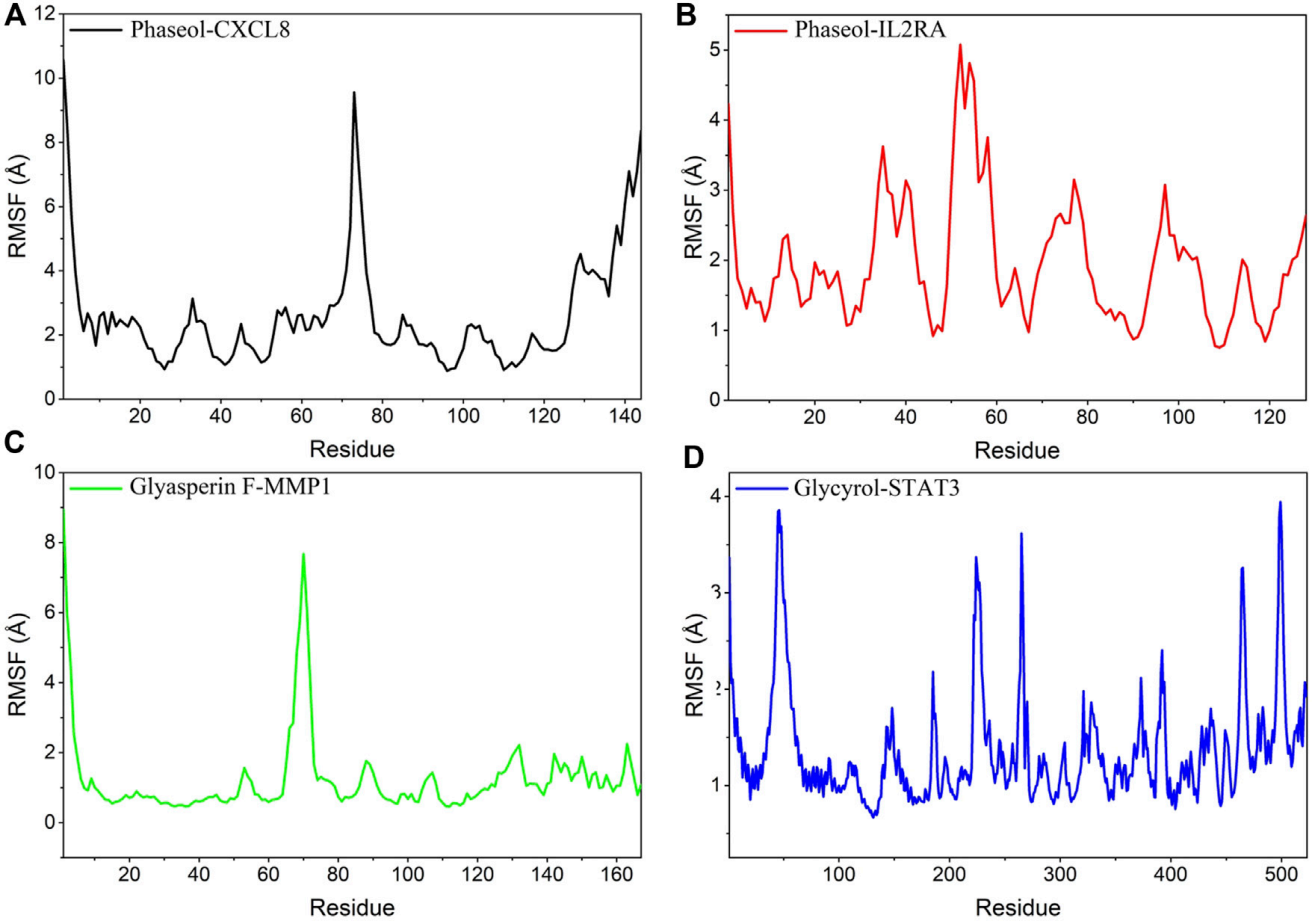
Changes in the stability of protein targets at the residue level. **(A)** CXCL8/Phaseol, **(B)** IL2RA/Phaseol, **(C)** MMP1/Glyasperin F, **(D)**STAT3/Glycyrol.

RMSF can respond to the flexibility of the protein during molecular dynamics simulation. Usually the protein flexibility decreases after the drug binds to the protein, which in turn achieves the effect of stabilizing the protein while exerting the enzymatic activity. The simulation results showed that the RMSF of proteins in MMP1/Glyasperin F, STAT3/Glycyrol were low. Especially for MMP1 protein, the RMSF of most of the dashed lines was below 2 Å, implying that the complex binding was more stable. In contrast, the RMSFs of the proteins in IL2RA/Phaseol and CXCL8/Phaseol were larger, suggesting that these two proteins were more flexible. The results are shown in Figure 9.

### Analysis of the radius of gyration

**FIGURE 10:**
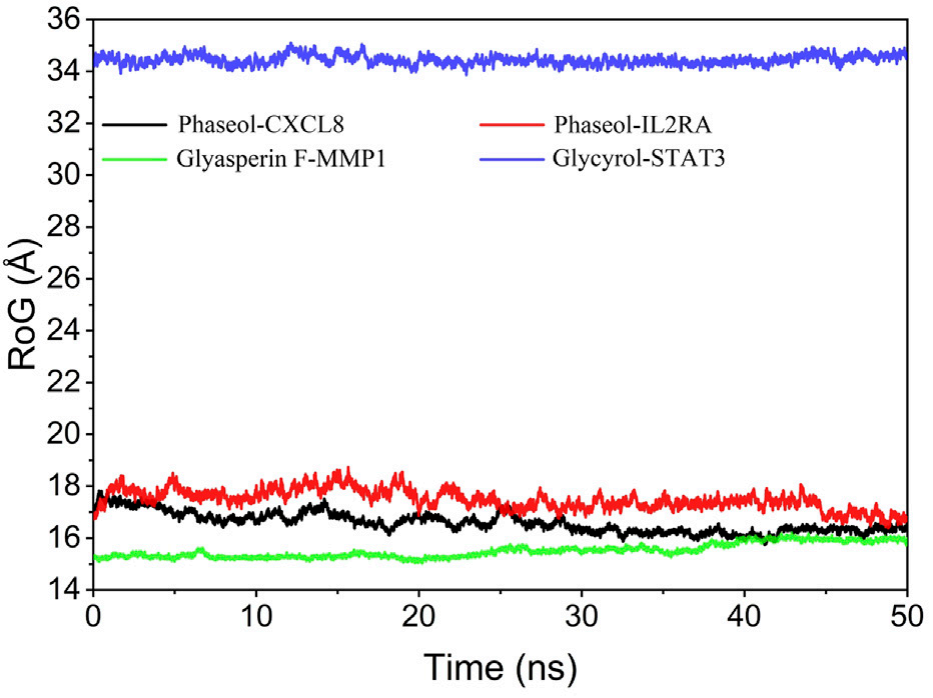
Analysis of protein folding state and overall conformation.

The radius of gyration can reflect the degree of compactness of the complex, and the size of fluctuation can be very intuitive to determine the compactness or system convergence. The fluctuations of the radius of gyration were MMP1/Glyasperin F, STAT3/Glycyrol, CXCL8/Phaseol, IL2RA/Phaseol from the largest to the smallest, respectively. The results are shown in Figure 10.

### Analysis of solvent accessible surface area

**FIGURE 11:**
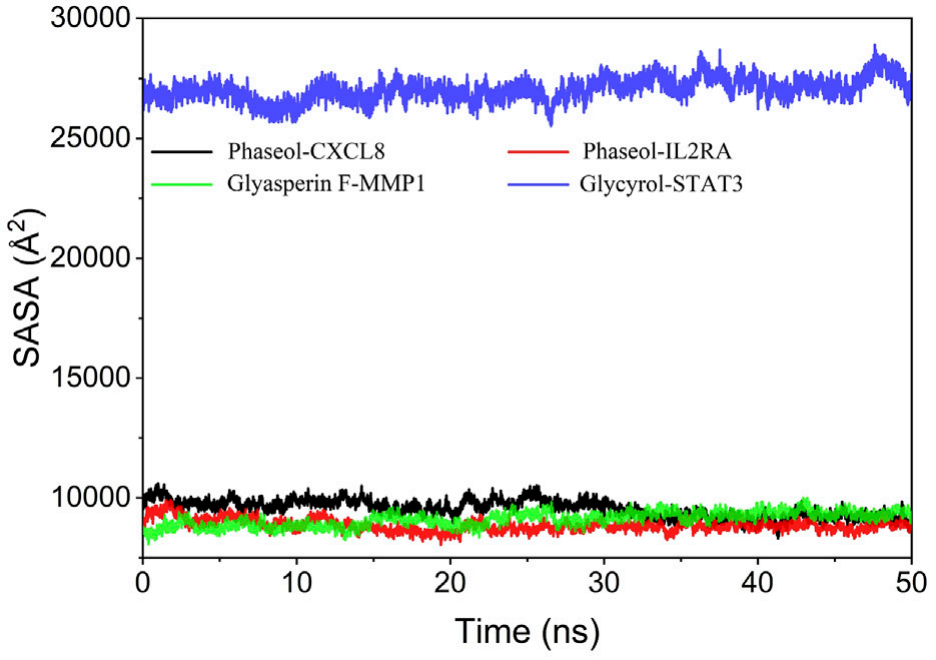
Analysis of Solvent Accessible Surface Area (SASA).

The Solvent Accessible Surface Area (SASA) is calculated as the interface surrounded by the solvent. The larger the area indicates that the complex can interact with the aqueous solution. In addition, the fluctuation of SASA reflects the exposure of the protein surface and the change of the buried area. The fluctuations of SASA suggested that MMP1/Glyasperin F, STAT3/Glycyrol, CXCL8/Phaseol fluctuated less and the SASA values were small. The results are shown in Figure 11.

## Discussion

In this study, we investigated the pharmacological mechanism of action of licorice for the treatment of COVID-19 by molecular docking and molecular dynamics simulation. It was found that the important active chemical components Phaseol in licorice may reduce inflammatory cell activation and inflammatory response by inhibiting the activation of CXCL8 and IL2RA; Glycyrol may act mainly on STAT3 to regulate cell proliferation and survival; And Glyasperin F may regulate cell growth by inhibiting the activation of MMP1, thereby reducing tissue damage and cell death caused by excessive inflammatory responses and promoting the growth of new tissues. Therefore, the active small molecules Phaseol, Glycyrol and Glyasperin F in licorice may act on CXCL8, IL2RA, STAT3, and MMP1 to treat COVID-19 by reducing tissue damage and inflammatory response.

### Analysis of bioinformatics results

In this study, Phaseol, Glycyrol and Glyasperin F in licorice may treat COVID-19 to reduce the inflammatory response and promote cell survival by acting on CXCL8, IL2RA, STAT3, and MMP1.

Phaseol may reduce inflammatory cell activation and inflammatory response by inhibiting the activation of CXCL8 and IL2RA. PPI analysis suggested that CXCL8 and IL2RA were closely associated with targets of inflammatory response regulation. GO analysis results suggested that CXCL8 was mainly involved in chemokine activity and interleukin eight receptor binding. KEGG pathway analysis identified IL2RA in pathways such as cellular senescence and MIF-mediated glucocorticoid regulation. The analysis showed that CXCL8 acted as a chemokine that attracts neutrophils, basophils and T cells, but not monocytes. It was also involved in neutrophil activation. The results of GO analysis suggested that IL2RA was mainly involved in drug binding and interleukin two binding. KEGG pathway analysis revealed IL2RA in pathways such as immune cell activation and tumor microenvironment regulation. The results suggested that IL2RA was involved in the regulation of immune tolerance by controlling the activity of regulatory T cells (TREG), which could regulate the inflammatory response by suppressing the activation and expansion of self-reactive T cells.

Glycyrol may act mainly on STAT3 to regulate cell proliferation and survival. PPI analysis suggested that STAT3 was closely associated with targets that regulate cell growth and apoptosis. GO analysis results suggested that STAT3 was mainly involved in DNA-binding transcription factor activity and sequence-specific DNA binding. KEGG pathway analysis identified STAT3 in pathways such as cellular senescence. The results of the analysis suggested that IL2RA was involved in the regulation of immune tolerance through the control of regulatory T cell (TREG) activity. TREG can regulate inflammatory responses by suppressing the activation and expansion of self-reactive T cells. The analysis showed that IL6 could participate in cell cycle regulation by regulating the transcriptional activity of STAT3, and STAT3 inhibited cellular autophagy by suppressing EIF2AK2/PKR activity.

Glyasperin F may regulate cell growth by inhibiting the activation of MMP1. PPI analysis suggested that MMP1 was closely associated with targets that regulate protein hydrolysis and processing. GO analysis results suggested that MMP1 was mainly involved in calcium binding and metallopeptidase activity. KEGG pathway analysis identified MMP1 in interleukin six family signaling and other pathways. The analysis showed that MMP1 was mainly involved in extracellular matrix breakdown in normal physiological processes (such as: embryonic development, reproduction and tissue remodeling) as well as in disease processes (such as: arthritis and metastasis).

However, the results of bioinformatics analysis could only predict the potential relationship between the drug and the key target. Therefore, this study further validated the mechanism of action of licorice for COVID-19 treatment using molecular docking and molecular dynamics.

### Analysis of molecular docking and molecular dynamics

Molecular docking simulations revealed strong affinity of drug active ingredients (such as: Phaseol, Glycyrol, and Glyasperin F) to protein targets (such as: CXCL8, IL2RA, STAT3, and MMP1). Molecular dynamics results suggested that the drug small molecules and protein complexes could maintain a very stable binding state and thus exert pharmacological effects in the treatment of COVID-19.

Phaseol was able to act stably on CXCL8 and IL2RA, and in particular CXCL8/Phaseol showed strong stability. Molecular docking showed that the binding energy of small molecule Phaseol to CXCL8 and IL2RA reached −8.9 and −8.3, respectively. Based on the trajectory of molecular dynamics simulations, we used the MMGBSA method to calculate the binding energy, which can more accurately reflect the binding mode of small molecules to target proteins. The binding free energy results showed −39.51 ± 2.06 kcal/mol and −20.12 ± 3.38 kcal/mol for CXCL8/Phaseol and IL2RA/Phaseol. In molecular dynamics simulations, the RMSD of both CXCL8/Phaseol and IL2RA/Phaseol gradually converged in the first 10 ns of the simulation and maintained stable fluctuations in subsequent simulations, implying increasing stability of the complex after binding. CXCL8/Phaseol binding results showed that the small molecule Phaseol interacted with E29 on the protein by hydrogen bonding and with V25, V27, V58, and I22 by hydrophobic interaction. The IL2RA/Phaseol binding results showed that the small molecule of drug interacted with Y119, E116, R117, T14, and E9 on the protein by hydrogen bonding, and with Y119, F121, F15, E9, and E116 by hydrophobic interaction, and the pi-pi conjugation occurred between Phaseol and F15.

The binding of Glycyrol to STAT3 was relatively stable and molecular docking showed that the binding energy of the small molecule to NLRP3 was −7.8. The free energy of binding results showed STAT3/Glycyrol to be −11.85 ± 1.06 kcal/mol. The RMSD fluctuations of STAT3/Glycyrol were all within 2 Å, implying a small movement of the STAT3/Glycyrol system. The STAT3/Glycyrol binding results indicated that the small molecule Glycyrol interacted with S611, E612, and S613 on the protein by hydrogen bonding, with P629 and S613 by hydrophobic interaction, and also with R609 by cation pi conjugation.

Glyasperin F bound to MMP1 could form a very stable complex. Molecular docking showed that the binding energy of small molecule Glyasperin F to MMP1 reached −9.8. The binding free energy results showed that MMP1/Glyasperin F was −43.70 ± 1.80 kcal/mol. The hydrogen bonding of MMP1/Glyasperin F was sparse at the late stage of molecular dynamics simulation, implying that hydrogen bonding was not the main force for its stability maintenance. The results of MMP1/Glyasperin F binding indicated that the small molecule interacted with A84 on the protein by hydrogen bonding and with H83, V115, L81, Y140, and H118 by hydrophobic interaction.

This study not only analyzed the relevant bioinformatics findings, but also used a supercomputer platform to simulate the microscopic evolution of complex systems of small molecule drugs and proteins through molecular dynamics. The computer simulations visualized the binding states of CXCL8/Phaseol, IL2RA/Phaseol, STAT3/Glycyrol and MMP1/Glyasperin F. The results of molecular dynamics simulations showed that the simulated binding of the four complexes could remain relatively stable.

Therefore, the results of this study can further explain the mechanism of action of active small molecules of licorice for the treatment of COVID-19 and related signaling pathways.

### Phaseol may reduce inflammatory cell activation and inflammatory response through CXCL8 and IL2RA

Phaseol is the active component derived from licorice. Phaseol was found to be closely associated with the IL6-STAT3 signaling pathway (Lu et al., 2020), and Phaseol could alleviate the inflammatory effects in lipopolysaccharide (LPS)-induced RAW264.7 cells (Li et al., 2017).

CXCL8 (also known as CXCL8) belongs to the elastin-like recombinant (ELR) CXC chemokine family (Liu et al., 2016). CXCL8 can be secreted by different cell types, including blood monocytes, alveolar macrophages, fibroblasts, endothelial cells, and epithelial cells (Ha et al., 2017). CXCL8 acts as a chemokine by directing neutrophils to the site of infection. Moreover, CXCL8 is also involved in pro-inflammatory signaling cascades along with other cytokines and plays a role in systemic inflammatory response syndrome (SIRS).

CXCL8 is a highly selective pro-inflammatory chemokine, and local and systemic elevations of CXCL8 have been found in various inflammatory diseases as well as in SIRS and sepsis (Haas et al., 2016). CXCL8 is barely detectable in the physiological state, but can be stimulated by pro-inflammatory cytokines such as tumor necrosis factor a (TNFa) and interleukin-1b (IL-1b) and mediated by the transcription factors NF-κB and activator protein-1 (AP-1), which can lead to a 10 to 100 fold upregulation of CXCL8 expression. The function of CXCL8 is mainly dependent on its interaction with specific cell surface G protein-coupled receptors (GPCR), CXCR1 and CXCR2 (Liu et al., 2016; Ha et al., 2017). CXCL8 contributes to the pathology of angiogenesis, fibrosis, infection, atherosclerosis, and tumor growth. Clinical studies have shown that elevated plasma levels of CXCL8 and other ELR-CXC chemokines can occur with acute indications such as arthritis, chronic obstructive pulmonary disease (COPD), asthma, cystic fibrosis, atherosclerosis, inflammatory bowel disease (IBD), psoriasis, and cancer, as well as acute indications such as reperfusion injury and acute respiratory distress syndrome (ARDS) (Cheng et al., 2017). Leukocyte recruitment is critical in many acute and chronic inflammatory diseases. Chemokines are key mediators of leukocyte recruitment during the inflammatory response, and the chemokine interleukin-8/CXCL8 is a classic neutrophil chemoattractant (Martínez-Burgo et al., 2019). CXCL8 inhibits the chemotactic response of neutrophils and suppresses the neutrophil-induced inflammatory response (Zhou et al., 2019). And CXCL8 promotes the activation and recruitment of macrophages and monocytes, which is a prerequisite for the shift from acute to chronic inflammation (Mohr et al., 2017). CXCL8 has been reported to recruit leukocytes from the blood into tissues during inflammation, and in turn, inflammation worsened by activated leukocytes can increase CXCL8 levels (Zhou et al., 2019). And it has been shown that monoammonium glycyrrhizinate (MAG) of licorice has anti-inflammatory properties. Mag inhibited the mRNA expression of TNF-α-induced chemokines (including CXCL8, CX3CL1, and CXCL16) in human dermal microvascular endothelial cell line (HMEC-1) cells in a dose-dependent manner and reduced the secretion of these chemokines (Cao et al., 2014).

IL2RA (also known as CD25) is a core component of the trimeric IL-2 receptor complex and plays a key role in mediating interleukin two immunomodulatory functions (Borysewicz-Sańczyk et al., 2020). IL2RA is a membrane protein that is involved in the regulation of immune tolerance by controlling the activity of regulatory T cells (TREG). Interleukin 2 (IL2) is a lymphocyte growth factor that plays an important role in the regulation of immune homeostasis as an essential self-tolerance regulator. It was found that cellular responsiveness to IL-2 directly depends on cellular expression of IL2RA, that IL-2 signaling increases with increased IL2RA expression, and that IL2RA directly affects binding stability in the IL-2/IL-2R complex (Buhelt et al., 2019). Plasma IL2RA levels were also found to be significantly elevated in COVID-19 patients (Galván-Peña et al., 2021; Sayah et al., 2021).

IL2RA is the receptor subunit that increases the affinity of the receptor for IL2 cytokines (Akman et al., 2021). Expression of IL2RA has been described at high levels on the surface of regulatory T cells (Tregs), a population of T cells with the ability to suppress self-reactive T cells. Further studies have shown that IL2RA plays a crucial role in sensitizing T cells to induce cell death (Borysewicz-Sańczyk et al., 2020). Changes in IL2RA expression may affect immune and inflammatory signaling cascade responses, which in turn affect CD4^+^ T cell differentiation and TReg cell suppressive activity (Asouri et al., 2020). IL2 signaling is involved in the differentiation and homeostasis of regulatory T cells (Tregs), and IL2 signaling is involved in the induction of cell growth and effector T cell proliferation (Zeebroeck et al., 2021). Pre-activation of IL-12, IL-15, and IL-18 was shown to upregulate IL2RA (CD25) expression (Akman et al., 2021). Further studies have shown that IL2RA plays a crucial role in sensitizing T cells to induce cell death.

Therefore, we suggested that Phaseol may reduce inflammatory cell activation and inflammatory response by acting on CXCL8 and IL2RA, thereby reducing tissue damage from excessive inflammatory response and alleviating the clinical symptoms of COVID-19.

### Glycyrol may affect cell proliferation and survival by regulating STAT3

Glycyrol exhibits a variety of biological effects, including antioxidant and anti-inflammatory effects and modulation of intrinsic immunity (Shin et al., 2011; Fu et al., 2014; Kim et al., 2020). It has been shown that Glycyrol-induced cell death is associated with apoptosis and autophagy, Glycyrol can bind to TOPK proteins and inhibit their kinase activity, leading to the activation of apoptotic signalling pathways (Xu and Kim, 2014; Lu et al., 2019).

STAT3 is a component of the acute phase response factor (APRF) complex activated by interleukin-6 (IL-6), and STAT3 plays a key role in many cellular processes such as cell growth and apoptosis by mediating the expression of multiple cellular stimuli (Hillmer et al., 2016). STAT3 is involved in regulating biological processes such as cell growth, differentiation and survival, inflammation and hematopoiesis (Gao et al., 2018; Liu et al., 2021; Zhao et al., 2021).

STAT3 is a latent transcription factor that mediates extracellular signals, such as cytokines and growth factors, by interacting with peptide receptors on the cell surface. STAT3 protein is transcriptionally activated mainly through tyrosine phosphorylation. Activated STAT3 dimers translocate to the nucleus and bind to sequence-specific DNA elements, thereby transcribing target genes (You et al., 2015). Recent studies have shown that STAT3 protein is expressed in CD4 T cells, T helper Th17 cells, Th1 and Th2 cells and that STAT3α isoforms may interact with proteins such as Probanin one to regulate pathological immune responses. The IL-6/JAK/STAT3 pathway is a major signaling pathway involved in regulating the inflammatory response in disease pathogenesis. JAK/STAT3 signaling promotes inflammation by regulating the development of innate lymphocytes in the immune response (Kang et al., 2021). STAT3 plays a central role in JAK/STAT signaling (You et al., 2015). IL-6 is the main stimulator of STAT3 *in vivo*, especially during inflammatory outbreaks. IL-6 signaling acts primarily through the JAK/STAT pathway, mainly through STAT3. Both of these factors can form IL-6 amplifiers that produce a cascade of amplifying effects associated with inflammation. This effect promotes various pro-inflammatory cytokines and chemokines, including IL-6, and recruits macrophages and lymphocytes, thereby enhancing the positive feedback loop formed by IL-6 and STAT3 (Luo et al., 2022). Licorice was found to reduce IL-6 levels, which is the main stimulator of STAT3 *in vivo*, especially during inflammatory outbreaks (Richard, 2021). Inhibition of STAT3 activity improved the pulmonary inflammatory response in LPS-induced acute lung injury (ALI) (Xu et al., 2020). And one study found a clinical therapeutic effect on lung inflammation by inhibiting STAT3 pathway (Zhao et al., 2016).

Therefore, we suggested that Glycyrol may act on STAT3 to regulate cell proliferation and survival, thereby reducing cell death due to inflammatory stimuli and promoting the growth of new tissue.

### Glyasperin F may regulate cell growth by affecting the activation of MMP1

Glyasperin F is an isoflavone compound, studies have found that Glyasperin F can inhibit the proliferation of lung cancer cells (Ngnintedo et al., 2016; Kuete et al., 2018).

MMP-1 is one of the most abundant enzymes in the family of matrix metalloproteinases (MMPs), which are mesenchymal collagenases secreted by a variety of cells including fibroblasts, endothelial and inflammatory cells (Gopal et al., 2016; Erdem et al., 2020). MMP-1 is capable of degrading type I, II, and III collagen, which plays a key role in extracellular matrix (ECM) remodeling in normal development and pathology (Affara et al., 2011).

MMP1 can be activated by several pro-inflammatory cytokines and growth factors and its expression is increased in alveolar epithelial cells during pulmonary fibrosis, and it inhibits mitochondrial respiration and oxidative stress, while promoting cell proliferation and migration (Lee et al., 2013). Various inflammatory factors (including CXCL8, IL-1β, and TNF-α) have been reported to contribute to the expression of MMP1 (Chen et al., 2019). MMP1 plays a clinically important role in inflammatory diseases and has been associated with many pathological processes, including wound healing, tumor metastasis and arthritis (Affara et al., 2011). Several reports suggest that MMP1 is indeed upregulated in patients suffering from diseases such as COPD and lung cancer (Carver et al., 2015). MMP-1 has been widely reported to lyse the extracellular matrix (ECM) and to promote angiogenesis. MMP1 was found to induce expression of vascular endothelial growth factor receptor 2 (VEGFR2) and endothelial cell proliferation, stimulate the serine/threonine protein kinase MARK2 and activate the transcription factor NF-κB for vascular remodeling and angiogenesis (Ng et al., 2022).

Many studies have found that licorice inhibited the high expression of matrix metalloproteinase-1 (MMP-1) and -3 (MMP-3) and down-regulated the expression of inflammatory cytokines such as IL-6, TNF-α, and IL-10. These findings strongly suggest that licorice regulates the abnormal expression of MMP-1 and MMP-3 mainly through its antioxidant and anti-inflammatory properties as well as (Kong et al., 2015; Gopal et al., 2016).

Therefore, we proposed that Glyasperin F may regulate cell growth by affecting the activation of MMP1, thereby promoting recovery of injured tissues.

### The mechanisms analysis of licorice in the treatment of corona virus disease 2019

The summary of the mechanisms analysis of licorice in the treatment of COVID-19 is shown in Graphical Abstract.

## Conclusion

This study explored the pharmacological mechanism of licorice for the treatment of COVID-19 by molecular docking and molecular dynamics simulations. We found that Phaseol in licorice may reduce inflammatory cell activation and inflammatory response by inhibiting the activation of CXCL8 and IL2RA; Glycyrol may regulate cell proliferation and survival by acting on STAT3. And Glyasperin F may regulate cell growth by inhibiting the activation of MMP1, thus reducing tissue damage and cell death caused by excessive inflammatory response and promoting the growth of new tissues.

## Data Availability

The datasets presented in this study can be found in online repositories. The names of the repository/repositories and accession number(s) can be found in the article/supplementary material.

## References

[B1] Abraham J. Florentine S. (2021). Licorice (Glycyrrhiza glabra) extracts-suitable pharmacological interventions for COVID-19? A review. Plants (Basel) 10, 2600. 10.3390/plants10122600 34961070 PMC8708549

[B2] Affara M. Dunmore B. J. Sanders D. A. Johnson N. Print C. G. Charnock-Jones D. S. (2011). MMP1 bimodal expression and differential response to inflammatory mediators is linked to promoter polymorphisms. BMC Genomics 12, 43. 10.1186/1471-2164-12-43 21244711 PMC3040839

[B3] Akman B. Hu X. Liu X. HatipoğluHua Y. Chen Y. Chan W. C. (2021). PRDM1 decreases sensitivity of human NK cells to IL2-induced cell expansion by directly repressing CD25 (IL2RA). J. Leukoc. Biol. 109, 901–914. 10.1002/JLB.2A0520-321RR 33145806 PMC8084872

[B4] Al-Shar'i N. A. Al-Balas Q. A. (2019). Molecular dynamics simulations of adenosine receptors: Advances, applications and trends. Curr. Pharm. Des. 25, 783–816. 10.2174/1381612825666190304123414 30834825

[B5] Asouri M. Rokni H. A. Sahraian M. A. Fattahi S. Motamed N. Doosti R. (2020). Analysis of single nucleotide polymorphisms in HLA-DRA, IL2RA, and HMGB1 genes in multiple sclerosis. Rep. Biochem. Mol. Biol. 9, 198–208. 10.29252/rbmb.9.2.199 33178870 PMC7603253

[B6] Borysewicz-Sańczyk H. Sawicka B. Wawrusiewicz-Kurylonek N. Głowińska-Olszewska B. Kadłubiska A. Gościk J. (2020). Genetic association study of IL2RA, IFIH1, and CTLA-4 polymorphisms with autoimmune thyroid diseases and type 1 diabetes. Front. Pediatr. 8, 481. 10.3389/fped.2020.00481 32974248 PMC7473350

[B7] Buhelt S. Søndergaard H. B. Oturai A. Ullum H. Essen M. R. Sellebjerg F. (2019). Relationship between multiple sclerosis-associated IL2RA risk allele variants and circulating T cell phenotypes in healthy genotype-selected controls Cells, 8. Cells, E634. 10.3390/cells8060634 31242590 PMC6628508

[B8] Burley S. K. Berman H. M. Kleywegt G. J. Markley J. L. Nakamura H. Velankar S. (2017). Protein data bank (PDB): The single global macromolecular structure archive. Methods Mol. Biol. 1607, 627–641. 10.1007/978-1-4939-7000-1_26 28573592 PMC5823500

[B9] Cao J. Li L. Xiong L. Wang C. Chen Y. Zhang X. (2022). Research on the mechanism of berberine in the treatment of COVID-19 pneumonia pulmonary fibrosis using network pharmacology and molecular docking. Phytomed. Plus. 2, 100252. 10.1016/j.phyplu.2022.100252 35403089 PMC8895682

[B10] Cao N. Chen T. Guo Z. Qin S. Li M. (2014). Monoammonium glycyrrhizate suppresses tumor necrosis factor-α induced chemokine production in HMEC-1 cells, possibly by blocking the translocation of nuclear factor-κB into the nucleus. Can. J. Physiol. Pharmacol. 92, 859–865. 10.1139/cjpp-2014-0022 25272089

[B11] Carver P. I. Anguiano V. D'Armiento J. M. Shiomi T. (2015). Mmp1a and Mmp1b are not functional orthologs to human MMP1 in cigarette smoke induced lung disease. Exp. Toxicol. Pathol. 67, 153–159. 10.1016/j.etp.2014.11.004 25497407 PMC4308467

[B12] Chen Y. Peng S. Cen H. Lin Y. Huang C. Chen Y. (2019). MicroRNA hsa-miR-623 directly suppresses MMP1 and attenuates IL-8-induced metastasis in pancreatic cancer. Int. J. Oncol. 55, 142–156. 10.3892/ijo.2019.4803 31115512 PMC6561617

[B13] Chen Y. Zheng Y. Fong P. Mao S. Wang Q. (2020). The application of the MM/GBSA method in the binding pose prediction of FGFR inhibitors. Phys. Chem. Chem. Phys. 22, 9656–9663. 10.1039/d0cp00831a 32328599

[B14] Cheng H. Yu H. Gordon J. R. Li F. Cheng J. (2017). Effects of K11R and G31P mutations on the structure and biological activities of CXCL8: Solution structure of human CXCL8 _(3-72)_ K11r/G31P. Molecules 22, 1229. 10.3390/molecules22071229 PMC6152285 28754019

[B15] Collier T. A. Piggot T. J. Allison J. R. (2020). Molecular dynamics simulation of proteins. Methods Mol. Biol. 2073, 311–327. 10.1007/978-1-4939-9869-2_17 31612449

[B16] Erdem J. s. Arnoldussen Y. J. Tajik S. Ellingsen D. G. Zienolddiny S. (2020). Effects of mild steel welding fume particles on pulmonary epithelial inflammation and endothelial activation. Toxicol. Ind. Health 36, 995–1001. 10.1177/0748233720962685 33025859 PMC7756071

[B17] Fernandes Q. Inchakalody V. P. Merhi M. Mestiri S. Taib N. Abo El-Ella D. M. (2022). Emerging COVID-19 variants and their impact on SARS-CoV-2 diagnosis, therapeutics and vaccines. Ann. Med. 54, 524–540. 10.1080/07853890.2022.2031274 35132910 PMC8843115

[B18] Filipe H. A. L. Loura L. M. S. (2022). Molecular Dynamics Simulations: Advances and Applications, 27.Molecules 10.3390/molecules27072105 PMC9000824 35408504

[B19] Fu Y. Zhou H. Wang S. Wei Q. (2014). Glycyrol suppresses collagen-induced arthritis by regulating autoimmune and inflammatory responses. PLoS One 9 (7), e98137. 10.1371/journal.pone.0098137 25036817 PMC4103760

[B20] Galván-Peña S. Leon J. Chowdhary K. Michelson D. A. Vijaykumar B. Yang L. (2021). Profound Treg perturbations correlate with COVID-19 severity. Proc. Natl. Acad. Sci. U. S. A. 118, e2111315118. 10.1073/pnas.2111315118 34433692 PMC8449354

[B21] Gao Y. Zhao H. Wang P. Wang J. Zou L. (2018). The roles of SOCS3 and STAT3 in bacterial infection and inflammatory diseases. Scand. J. Immunol. 88, e12727. 10.1111/sji.12727 30341772

[B22] Gomaa A. A. Abdel-Wadood Y. A. (2021). The potential of glycyrrhizin and licorice extract in combating COVID-19 and associated conditions. Phytomed. Plus. 1, 100043. 10.1016/j.phyplu.2021.100043 35399823 PMC7886629

[B23] Gopal S. K. Greening D. W. Zhu H. Simpson R. J. Mathias R. A. (2016). Transformed MDCK cells secrete elevated MMP1 that generates LAMA5 fragments promoting endothelial cell angiogenesis. Sci. Rep. 6, 28321. 10.1038/srep28321 27324842 PMC4914959

[B24] Ha H. Debnath B. Neamati N. (2017). Role of the CXCL8-CXCR1/2 Axis in cancer and inflammatory diseases. Theranostics 7, 1543–1588. 10.7150/thno.15625 28529637 PMC5436513

[B25] Haas M. Kaup F. J. Neumann S. (2016). Canine pyometra: A model for the analysis of serum CXCL8 in inflammation. J. Vet. Med. Sci. 78, 375–381. 10.1292/jvms.15-0415 26522810 PMC4829503

[B26] Hillmer E. J. Zhang H. Li H. S. Watowich S. S. (2016). STAT3 signaling in immunity. Cytokine Growth Factor Rev. 31, 1–15. 10.1016/j.cytogfr.2016.05.001 27185365 PMC5050093

[B27] Kang D. Sp N. En C. Rugamba A. Jing X. Jing R. (2021). Non-toxic sulfur inhibits LPS-induced inflammation by regulating TLR-4 and JAK2/STAT3 through IL-6 signaling. Mol. Med. Rep. 24, 485. 10.3892/mmr.2021.12124 33907855 PMC8127030

[B28] Kim Y. Shrestha R. Kim S. Kim J. A. Lee J. Jeong T. C. (2020). *In vitro* characterization of Glycyrol metabolites in human liver microsomes using HR-resolution MS spectrometer coupled with tandem mass spectrometry. Xenobiotica. 50 (4), 380–388. 10.1080/00498254.2019.1636418 31233374

[B29] Kong S. Chen H. Yu X. Zhang X. Feng X. Kang X. (2015). The protective effect of 18β-Glycyrrhetinic acid against UV irradiation induced photoaging in mice. Exp. Gerontol. 61, 147–155. 10.1016/j.exger.2014.12.008 25498537

[B30] Kuete V. Ngnintedo D. Fotso G. W. Karaosmanoğlu O. Ngadjui B. T. Keumedjio F. (2018). Cytotoxicity of seputhecarpan D, thonningiol and 12 other phytochemicals from African flora towards human carcinoma cells. BMC Complement. Altern. Med. 18 (1), 36. 10.1186/s12906-018-2109-9 29378558 PMC5789597

[B31] Larini L. Mannella R. Leporini D. (2007). Langevin stabilization of molecular-dynamics simulations of polymers by means of quasisymplectic algorithms. J. Chem. Phys. 126, 104101. 10.1063/1.2464095 17362055

[B32] Lee K. M. Shim H. Lee G. S. Park I. H. Lee O. S. Lim S. C. (2013). Chitin from the extract of cuttlebone induces acute inflammation and enhances MMP1 expression. Biomol. Ther. 21, 246–250. 10.4062/biomolther.2013.036 PMC3830125 24265872

[B33] Lee T. S. Allen B. K. Giese T. J. Guo Z. Li P. Lin C. (2020). Alchemical binding free energy calculations in AMBER20: Advances and best practices for drug discovery. J. Chem. Inf. Model. 60, 5595–5623. 10.1021/acs.jcim.0c00613 32936637 PMC7686026

[B34] Li H. Yoon J. H. Won H. J. Ji H. S. Yuk H. J. Park K. H. (2017). Isotrifoliol inhibits pro-inflammatory mediators by suppression of TLR/NF-κB and TLR/MAPK signaling in LPS-induced RAW264.7 cells. Int. Immunopharmacol. 45, 110–119. 10.1016/j.intimp.2017.01.033 28192731

[B35] Li J. Xu D. Wang L. Zhang M. Zhang G. Li E. (2021). Glycyrrhizic acid inhibits SARS-CoV-2 infection by blocking spike protein-mediated cell attachment. Molecules 26, 6090. 10.3390/molecules26206090 34684671 PMC8539771

[B36] Liu Q. Li A. Tian Y. Wu D. Liu Y. Li T. (2016). The CXCL8-CXCR1/2 pathways in cancer. Cytokine Growth Factor Rev. 31, 61–71. 10.1016/j.cytogfr.2016.08.002 27578214 PMC6142815

[B37] Liu Y. Liao S. Bennett S. Tang H. Song D. Wood D. (2021). STAT3 and its targeting inhibitors in osteosarcoma. Cell Prolif. 54, e12974. 10.1111/cpr.12974 33382511 PMC7848963

[B38] Lu S. Ye L. Yin S. Zhao C. Yan M. Liu X. (2019). Glycyrol exerts potent therapeutic effect on lung cancer via directly inactivating T-LAK cell-originated protein kinase. Pharmacol. Res. 147, 104366. 10.1016/j.phrs.2019.104366 31377221

[B39] Lu X. Wu X. Jing L. Tao L. Zhang Y. Huang R. (2020). Network pharmacology analysis and experiments validation of the inhibitory effect of JianPi Fu recipe on colorectal cancer LoVo cells metastasis and growth. Evid. Based. Complement. Altern. Med. 2020, 4517483. 10.1155/2020/4517483 PMC7399765 32774415

[B40] Luo W. Ding R. Guo X. Zhan T. Tang T. Fan R. (2022). Clinical data mining reveals Gancao-Banxia as a potential herbal pair against moderate COVID-19 by dual binding to IL-6/STAT3. Comput. Biol. Med. 145, 105457. 10.1016/j.compbiomed.2022.105457 35366469 PMC8957363

[B41] Maier J. A. Martinez C. Kasavajhala K. Wickstrom L. Hauser K. E. Simmerling C. (2015). ff14SB: Improving the accuracy of protein side chain and backbone parameters from ff99SB. J. Chem. Theory Comput. 11, 3696–3713. 10.1021/acs.jctc.5b00255 26574453 PMC4821407

[B42] Majumder J. Minko T. (2021). Recent developments on therapeutic and diagnostic approaches for COVID-19. Aaps J. 23, 14. 10.1208/s12248-020-00532-2 33400058 PMC7784226

[B43] Martínez-Burgo B. Cobb S. L. Pohl E. Kashanin D. Paul T. Kirby J. A. (2019). A C-terminal CXCL8 peptide based on chemokine-glycosaminoglycan interactions reduces neutrophil adhesion and migration during inflammation. Immunology 157, 173–184. 10.1111/imm.13063 31013364 PMC7662424

[B70] Mithun R. Ismail C. Johra K. Mohammad A. A. Mohammad N. M. Rohitash Y. (2022). Identification of bioactive molecules from Triphala (Ayurvedic herbal formulation) as potential inhibitors of SARS-CoV-2 main protease (Mpro) through computational investigations. J. King Saud Univ. Sci. 34 (3), 101826. 10.1016/j.jksus.2022.101826 35035181 PMC8744360

[B44] Mohr T. Haudek-Prinz V. Slany A. Grillari J. Micksche M. Gerner C. (2017). Proteome profiling in IL-1β and VEGF-activated human umbilical vein endothelial cells delineates the interlink between inflammation and angiogenesis. PLoS One 12, e0179065. 10.1371/journal.pone.0179065 28617818 PMC5472280

[B45] Nam K. H. (2021). Room-temperature structure of xylitol-bound glucose isomerase by serial crystallography: Xylitol binding in the M1 site induces release of metal bound in the M2 site. Int. J. Mol. Sci. 22, 3892. 10.3390/ijms22083892 33918749 PMC8070043

[B46] Ng L. Wong S. K. Huang Z. Lam C. S. Chow A. K. Foo D. C. (2022). CD26 induces colorectal cancer angiogenesis and metastasis through CAV1/MMP1 signaling. Int. J. Mol. Sci. 23, 1181. 10.3390/ijms23031181 35163100 PMC8835326

[B47] Ng S. L. Khaw K. Y. Ong Y. S. Goh H. P. Kifli N. Teh S. P. (2021). Licorice: A potential herb in overcoming SARS-CoV-2 infections. J. Evid. Based. Integr. Med. 26, 2515690x21996662. 10.1177/2515690X21996662 PMC8020229 33787349

[B48] Ngnintedo D. Fotso G. W. Kuete V. Nana F. Sandjo L. P. Karaosmanoğlu O. (2016). Two new pterocarpans and a new pyrone derivative with cytotoxic activities from ptycholobium contortum (N.E.Br.) brummitt (leguminosae): Revised NMR assignment of mundulea lactone. Chem. Cent. J. 10, 58. 10.1186/s13065-016-0204-x 28316643 PMC5050614

[B49] Ochani R. Asad A. Yasmin F. Shaikh S. Khalid H. Batra S. (2021). COVID-19 pandemic: From origins to outcomes. A comprehensive review of viral pathogenesis, clinical manifestations, diagnostic evaluation, and management. Infez. Med. 29, 20–36. 33664170

[B50] Pan B. Fang S. Zhang J. Pan Y. Liu H. Wang Y. (2020). Chinese herbal compounds against SARS-CoV-2: Puerarin and quercetin impair the binding of viral S-protein to ACE2 receptor. Comput. Struct. Biotechnol. J. 18, 3518–3527. 10.1016/j.csbj.2020.11.010 33200026 PMC7657012

[B51] Rai P. Kumar B. K. Deekshit V. K. Karunasagar I. Karunasagar I. (2021). Detection technologies and recent developments in the diagnosis of COVID-19 infection. Appl. Microbiol. Biotechnol. 105, 441–455. 10.1007/s00253-020-11061-5 33394144 PMC7780074

[B52] Ravindranath P. A. Forli S. Goodsell D. S. Olson A. J. Sanner M. F. (2015). AutoDockFR: Advances in protein-ligand docking with explicitly specified binding site flexibility. PLoS Comput. Biol. 11, e1004586. 10.1371/journal.pcbi.1004586 26629955 PMC4667975

[B53] Richard S. A. (2021). Exploring the pivotal immunomodulatory and anti-inflammatory potentials of glycyrrhizic and glycyrrhetinic acids. Mediat. Inflamm. 2021, 6699560. 10.1155/2021/6699560 PMC7808814 33505216

[B55] Sayah W. Berkane I. Guermache I. Sabri M. Lakhal F. Z. Rahali S. Y. (2021). Interleukin-6, procalcitonin and neutrophil-to-lymphocyte ratio: Potential immune-inflammatory parameters to identify severe and fatal forms of COVID-19. Cytokine 141, 155428. 10.1016/j.cyto.2021.155428 33550165 PMC7834734

[B56] Shin E. M. Kim S. Merfort I. Kim Y. S. (2011). Glycyrol induces apoptosis in human Jurkat T cell lymphocytes via the Fas-FasL/caspase-8 pathway. Planta Med. 77 (3), 242–247. 10.1055/s-0030-1250260 20717871

[B57] Xie R. Lin Z. Zhong C. Li S. Chen B. Wu Y. (2021). Deciphering the potential anti-COVID-19 active ingredients in Andrographis paniculata (Burm. F.) Nees by combination of network pharmacology, molecular docking, and molecular dynamics. RSC Adv. 11, 36511–36517. 10.1039/d1ra06487h 35494378 PMC9043438

[B58] Xiong H. Dong Z. Lou G. Gan Q. Wang J. Huang Q. (2020). Analysis of the mechanism of Shufeng Jiedu capsule prevention and treatment for COVID-19 by network pharmacology tools. Eur. J. Integr. Med. 40, 101241. 10.1016/j.eujim.2020.101241 33520015 PMC7836709

[B59] Xu M. Y. Kim Y. S. (2014). Antitumor activity of Glycyrol via induction of cell cycle arrest, apoptosis and defective autophagy. Food Chem. Toxicol. 74, 311–319. 10.1016/j.fct.2014.10.023 25445757

[B60] Xu S. Pan X. Mao L. Pan H. Xu W. Hu Y. (2020). Phospho-Tyr705 of STAT3 is a therapeutic target for sepsis through regulating inflammation and coagulation. Cell Commun. Signal. 18, 104. 10.1186/s12964-020-00603-z 32641132 PMC7341624

[B61] Yang R. Wang L. Yuan B. Liu Y. (2015). The pharmacological activities of licorice. Planta Med. 81, 1654–1669. 10.1055/s-0035-1557893 26366756

[B62] Yi Y. Li J. Lai X. Zhang M. Kuang Y. Bao Y. O. (2022). Natural triterpenoids from licorice potently inhibit SARS-CoV-2 infection. J. Adv. Res. 36, 201–210. 10.1016/j.jare.2021.11.012 35116174 PMC8620242

[B63] You L. Wang Z. Li H. Shou J. Jing Z. Xie J. (2015). The role of STAT3 in autophagy. Autophagy 11, 729–739. 10.1080/15548627.2015.1017192 25951043 PMC4509450

[B64] Zeebroeck L. V. Hornero R. A. Côrte-Real B. F. Hamad I. Meissner T. B. Kleinewietfeld M. (2021). Fast and efficient genome editing of human FOXP3(+) regulatory T cells. Front. Immunol. 12, 655122. 10.3389/fimmu.2021.655122 34408743 PMC8365355

[B65] Zhang Q. H. Huang H. Z. Qiu M. Wu Z. F. Xin Z. C. Cai X. F. (2021). Traditional uses, pharmacological effects, and molecular mechanisms of licorice in potential therapy of COVID-19. Front. Pharmacol. 12, 719758. 10.3389/fphar.2021.719758 34899289 PMC8661450

[B66] Zhao J. Liu X. Chen Y. Zhang L. S. Zhang Y. R. Ji D. R. (2021). STAT3 promotes schistosome-induced liver injury by inflammation, oxidative stress, proliferation, and apoptosis signal pathway. Infect. Immun. 89, e00309-20. 10.1128/IAI.00309-20 33257536 PMC8097265

[B67] Zhao J. Yu H. Liu Y. Gibson S. A. Yan Z. Xu X. (2016). Protective effect of suppressing STAT3 activity in LPS-induced acute lung injury. Am. J. Physiol. Lung Cell. Mol. Physiol. 311, L868–L880. 10.1152/ajplung.00281.2016 27638904 PMC5130536

[B69] Zhou Y. Xu W. Zhu H. (2019). CXCL8((3-72)) K11R/G31P protects against sepsis-induced acute kidney injury via NF-κB and JAK2/STAT3 pathway. Biol. Res. 52, 29. 10.1186/s40659-019-0236-5 31084615 PMC6513525

